# SurvExpress: An Online Biomarker Validation Tool and Database for Cancer Gene Expression Data Using Survival Analysis

**DOI:** 10.1371/journal.pone.0074250

**Published:** 2013-09-16

**Authors:** Raul Aguirre-Gamboa, Hugo Gomez-Rueda, Emmanuel Martínez-Ledesma, Antonio Martínez-Torteya, Rafael Chacolla-Huaringa, Alberto Rodriguez-Barrientos, José G. Tamez-Peña, Victor Treviño

**Affiliations:** Cátedra de Bioinformática, Tecnológico de Monterrey, Monterrey, Nuevo León, México; Queen Elizabeth Hospital, Hong Kong

## Abstract

Validation of multi-gene biomarkers for clinical outcomes is one of the most important issues for cancer prognosis. An important source of information for virtual validation is the high number of available cancer datasets. Nevertheless, assessing the prognostic performance of a gene expression signature along datasets is a difficult task for Biologists and Physicians and also time-consuming for Statisticians and Bioinformaticians. Therefore, to facilitate performance comparisons and validations of survival biomarkers for cancer outcomes, we developed **SurvExpress**, a cancer-wide gene expression database with clinical outcomes and a web-based tool that provides survival analysis and risk assessment of cancer datasets. The main input of SurvExpress is only the biomarker gene list. We generated a cancer database collecting more than 20,000 samples and 130 datasets with censored clinical information covering tumors over 20 tissues. We implemented a web interface to perform biomarker validation and comparisons in this database, where a multivariate survival analysis can be accomplished in about one minute. We show the utility and simplicity of SurvExpress in two biomarker applications for breast and lung cancer. Compared to other tools, SurvExpress is the largest, most versatile, and quickest free tool available. SurvExpress web can be accessed in http://bioinformatica.mty.itesm.mx/SurvExpress (a tutorial is included). The website was implemented in JSP, JavaScript, MySQL, and R.

## Introduction

Cancer causes millions of deaths around the world. To improve treatments, several biomarkers have been proposed for risk prognosis and treatment response. Recent published biomarkers in many types of cancer contain numerous genes and are mainly based on gene expression. They have been generated using microarray profiling and lately by RNA-Seq technologies. Often, identified biomarkers are developed to a specific cancer tissue and subtypes. In breast cancer, for example, more than 40 biomarkers have been proposed containing between 3 and 512 genes and whose prognostic or predictive performance depends on therapy, hormone receptor status, and the number of genes [1], [2]. On the other hand, assessing the performance of proposed biomarkers in different populations or evaluating competing biomarkers are difficult tasks even though hundreds of public datasets are available. The main limitations are the time and resources needed for acquiring, processing, normalizing, filtering, and statistical modeling of large gene expression datasets. This is important since several of the reasons involved in the failure of biomarkers in clinical trials are related to data analysis [3]. For the analysis of biomarkers, tools as ITTACA, KMPlot, RecurrenceOnline, bc-GeneExMiner, GOBO, and PrognoScan have been proposed [1], [4]–[9]. However, these tools have serious restrictions (Table 1), complicating and limiting the assessment of multi-gene biomarkers in cancer. Some of the main limitations include considering just one gene at the time or a specific set of genes; focusing on breast or ovarian cancer datasets or to a particular Affymetrix gene expression platform; requiring the upload of Affymetrix gene expression data (.CEL files); and using a single quantity per gene even though some microarray platforms provide more probesets.

**Table 1:** Comparison of survival analysis tools.

Tool	Genes Per Analysis	Tissues	Datasets	Samples	Input	Risk Groups
**KMPlot** *	1	2	>30	4,441	AffyID	Quartiles
**ITTACA** **	1	7	23§	>739	Genes, Groups	User
**Recurrence Online**	Specific	1	–	–	CEL Files	Specific
**bc-GenExMiner**	1	1	21	3,414	Genes	Median
**PrognoScan**	1	14	74§	8,626	Genes	Algorithm
**GOBO** *	Multiple	1	10	1,881	Genes	Classifier
**SurvExpress**	Multiple	22	142	21,051	Genes	Various***

Comparison of survival analysis tools in terms of genes, databases, input, and risk group generation.

* Data includes only Affymetrix microarrays.

** Only few of the datasets have survival data.

*** SurvExpress risk groups can be generated based on the Cox fitting, an optimization algorithm, or user-specified weights.

§ Only including datasets with clinical outcome information.

To solve these issues and to facilitate performance comparisons and validations of prognostic and predictive biomarkers for cancer outcomes, we developed SurvExpress. SurvExpress is a comprehensive gene expression database and web-based tool providing survival analysis and risk assessment in cancer datasets using a biomarker gene list as input. The tool is available in http://bioinformatica.mty.itesm.mx/SurvExpress. The tool includes a tutorial that describes the analysis options, plots, tables, key concepts related to survival analysis, and representative methods to identify biomarkers from gene expression data.

## Materials and Methods

### Database Acquisition

Datasets were obtained mainly from GEO (http://www.ncbi.nlm.nih.gov/geo/) and TCGA (https://tcga-data.nci.nih.gov) after searching for keywords related to cancer, survival, and gene expression technologies. Additionally, a few were obtained from author’s websites and from ArrayExpress (http://www.ebi.ac.uk/arrayexpress/). The data source used is shown in the web interface. We favored cancer types above two different cohorts and datasets containing survival data over 30 samples in which censoring indicator and time to death, recurrence, relapse, or metastasis were provided. Clinical data was provided by dataset authors via personal email when not available online in corresponding repositories. Datasets were annotated from provider files as found up to September 2012, and were quantile-normalized and log2 transformed when needed. From TCGA, all datasets were obtained at the gene level (level 3). RNA-Seq counts data were log2 transformed. In some cancer types where many datasets were found for the same gene expression platform, we also provide a merged meta-base. In meta-bases, datasets were quantile normalized; probesets means were equalized conserving the standard deviation by each cohort; and datasets were merged by probeset id. At the moment we provide meta-bases for breast, lung, and ovarian cancer. To facilitate gene searches and conversions between gene identifiers, human gene information was used and obtained from the NCBI FTP site (ftp://ftp.ncbi.nih.gov/gene/DATA/GENE_INFO/Mammalia/Homo_sapiens.gene_info.gz). To simplify the user interface, datasets were grouped by related organ or tissue using disease ontologies [10].

### Web Interface Implementation

**Figure 1:**
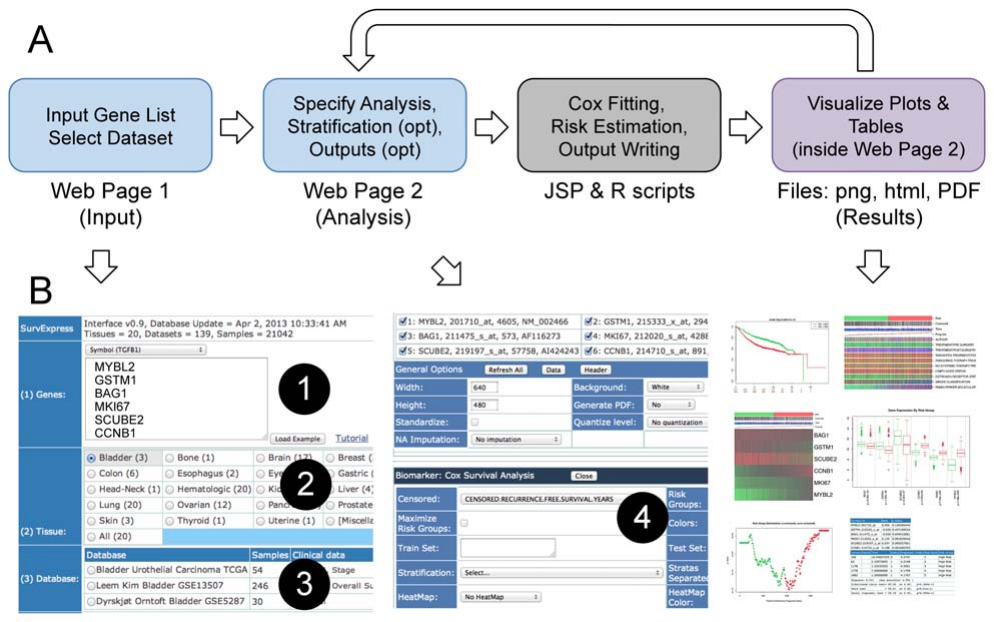
Overview of the SurvExpress web tool. Panel A shows a schematic diagram of the SurvExpress workflow while Panel B shows snapshots of the interfaces tagging the required input fields. In the first *Input* web page, the user can paste the list of genes (tagged with the number 1, which can be symbols, entrez gene identifier and others identifiers) and choose the dataset from around 140 available datasets (tagged with 2 and 3). SurvExpress validates and searches the genes and dataset to show the *Analysis* web page where the user selects the censored outcome (tag 4) and visualizes the results (right-bottom expanded in Figure 2). The whole process can be achieved in less than one minute for a sensible number of genes.

Two simple and lightweight HTML user interfaces based on java server pages, JavaScript, R, Ajax, Apache, and MySQL were implemented (Figure 1A). In the *Input* page, users introduce the gene list based on NCBI compatible gene identifiers (official symbol, Entrez, Ensembl, HGNC, or others) and select the target dataset. Users can also choose how to treat genes having more than one probe. The *Analysis* page extracts the dataset rows related to genes in the biomarker and delivers a web interface. Then, users can assess the biomarker in a variety of ways, including switching on and off specific genes, stratifying samples by available clinical information (e.g. stage, grade, age, biochemical results, and mutation status), specifying training and test samples, and weighting genes instead of using the Cox fitting. The results are displayed in common and flexible publication-ready plots and tables within the *Analysis* page. A PDF version of the results can also be obtained.

### Prognostic Index Estimation

The prognostic index (PI), also known as the risk score, is commonly used to generate risk groups. The PI is known as the linear component of the Cox model [11], PI = *β_1_x_1_+ β_2_x_2_+...+β_p_x_p_* where *x_i_* is the expression value and the *β_I_* can obtained from the Cox fitting. Each *β_I_* can be interpreted as a risk coefficient. SurvExpress implements two procedures to estimate the *β* coefficients. The first procedure is the classical Cox model where all genes are included in a unique model. The fitting is performed in R (http://cran.r-project.org) using the *survival* package. In the second procedure, the user can specify a weight for each gene instead of using the values from the Cox fitting. Such option is useful to make comparisons with biomarkers computed with mathematical models other than Cox.

### Risk Estimation

SurvExpress implements two methods to generate risk groups. The first method (default) generates the risk groups splitting the ordered PI (higher values for higher risk) by the number of risk groups leaving equal number of samples in each group. For two risk groups, this is equivalent to split the PI by the median. The second method to produce risk groups uses an optimization algorithm from the ordered PI. Briefly, for two groups, a log-rank test is performed along all values of the arranged PI. Then, the algorithm chooses the split point where the p-value is minimum. This procedure is generalized for more than two groups repeatedly optimizing one risk group at the time until no changes are observed. Details of this procedure are described in the tutorial provided in SurvExpress web site.

### Outputs

**Figure 2:**
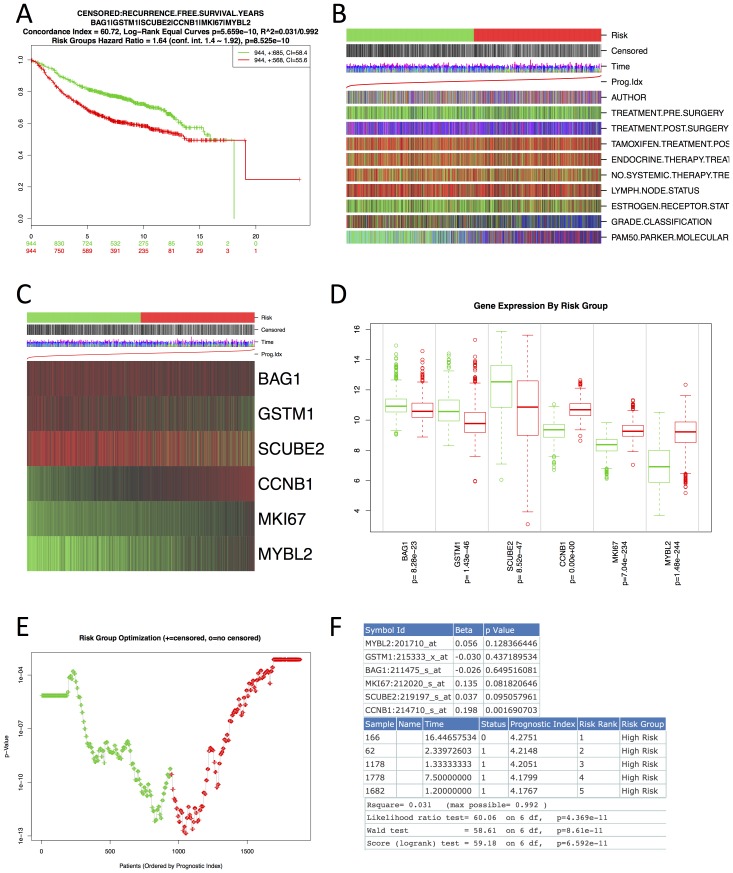
Common outputs of the SurvExpress Results page. This figure shows the results from a breast cancer meta-base included in SurvExpress. Panel A shows the Kaplan-Meier curve for risk groups, concordance index, and p-value of the log-rank testing equality of survival curves. Panel B shows clinical information available related to risk group, prognostic index, and outcome data. Panel C shows a heat map representation of the gene expression values. Panel D shows a box plot across risk groups, including the p-value testing for difference using t-test (or f-test for more than two groups). Panel E shows the relation between risk groups and prognostic index. Panel F shows fragments of tables with the summary of the Cox fitting and the prognostic indexes. Details are provided in SurvExpress Tutorial.

The outputs included correspond to common metrics and plots used to assess the performance of survival data. An example of the outputs generated by SurvExpress is shown in Figure 2. Panel A shows the Kaplan-Meier plots by risk group, the log-rank test of differences between risk groups, the hazard-ratio estimate, and the concordance indexes, which estimate the probability that subjects with a higher risk will experience the event after subjects with a lower risk [12]. Panel B displays a visual association of available clinical information to risk groups. Panel C illustrates a heat map of gene expression values. Panel D shows box plots of gene expression values across gene groups together with the p-value of the corresponding difference. Panel E demonstrates the risk group optimization plot. Panel F shows fragments of the tables for the beta coefficients including corresponding Cox p-values, prognostic index per sample, and Cox fitting information from the *survival* package in R. Other advanced plots are also available in the tutorial provided in SurvExpress. Other ‘advanced plots’ include SurvivalROC that estimates time-dependent sensitivities and specificities for survival risk groups [13] but needs a few minutes to compute. Additional plots, details and interpretations of the outputs are described in the tutorial provided in the SurvExpress web site.

## Results and Applications

### Database

Although data collection will continue, to date we have collected around 20,000 cancer samples distributed in 140 datasets covering more than 20 tissues (Table 2). The main limitation to include more datasets was that the absence of censoring information in repositories. Nevertheless, the SurvExpress collection surpasses that of similar tools in terms of tissue coverage, number of samples, multivariate predictor estimation, and functionality (Table 1). From the 20 cancer types, the most represented by their number of datasets were breast, hematologic, lung, brain, and ovarian, reaching around 70% of the database collection. It is surprising that most of the existing tools are mainly concentrated in breast cancer even though a similar number of datasets is available for other cancer types. Consequently, one of the immediate advantages of SurvExpress is the availability to perform powerful analysis for these highly studied types of cancers. In addition, SurvExpress will allow the validation of biomarkers in cancer types that have not been considered by other tools such as kidney, liver, gastrointestinal, pancreatic, bone, head and neck, and uterine. In the web interface, we also encourage users to suggest or send data to increase cancer and dataset coverage.

**Table 2:** Current content of the SurvExpress database per cancer type.

Cancer Type	Datasets
Breast	25
Hematologic	20
Lung	19
Brain	17
Ovarian	12
Colon	6
Kidney	6
Liver	4
Prostate	4
Gastrointestinal	4
Bladder	3
Head & Neck	3
Skin	3
[Miscellaneous]	3
Esophagus	2
Eye	2
Pancreas	2
Bone	1
Oral	1
Stomach	1
Uterine	1

### Web Interface

The two web interfaces comprise three sections: *Input, Analysis* and *Results* (Figure 1B). The *Input* page is easily operated typing or pasting a list of genes and specifying the target dataset (numbers 1 to 3 in Figure 1B). It also includes a link to the tutorial that describes all options and provides comprehensive interpretations of the outputs. The subsequent *Analysis* and *Result* page is obtained in a few seconds (about 1 second per gene and 200 samples). In the *Analysis* section, the user specifies the outcome of the selected dataset in which the analysis will be performed (number 4 in Figure 1B). The *Results* section (Figure 2) is obtained few seconds after submitting an analysis. This section includes outputs such as Kaplan-Meier curves for risk groups, visual comparison of the clinical information to risk groups, a heat map of the gene expression values, box plots of the gene expression per gene and risk group, a plot of the risk group optimization process, tables of the Cox coefficients, prognostic indexes, and Cox fitting information, and a link to obtain the R scripts used.

### Validation and Applications

Because of limitations in other tools, multi-gene comparisons across tools were not possible. Still, SurvExpress can provide similar results to other tools when one gene only is used. Nevertheless, to assess the functionality and estimations of SurvExpress, we performed two analyses evaluating the performance of well-known and proposed prognostic biomarkers. We used the OncotypeDX biomarker for recurrence in breast cancer and two published biomarkers for lung cancer survival.

#### OncotypeDX biomarker for breast cancer

**Figure 3:**
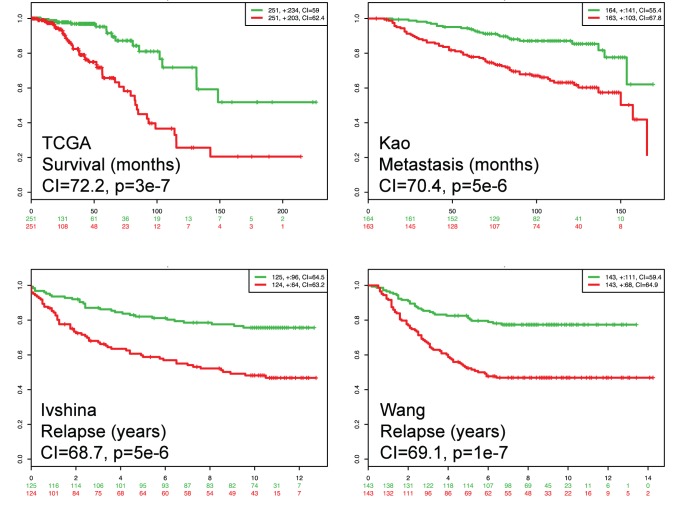
Kaplan-Meier curves and performance of the OncoTypeDX biomarker in four datasets. Censoring samples are shown as “+” marks. Horizontal axis represents time to event. Dataset, outcome event, time scale, concordance index (CI), and p-value of the log-rank test are shown. Red and Green curves denote High- and Low-risk groups respectively. The red and green numbers below horizontal axis represent the number of individuals not presenting the event of the corresponding risk group along time. The number of individuals, the number of censored, and the CI of each risk group are shown in the top-right insets.

**Figure 4:**
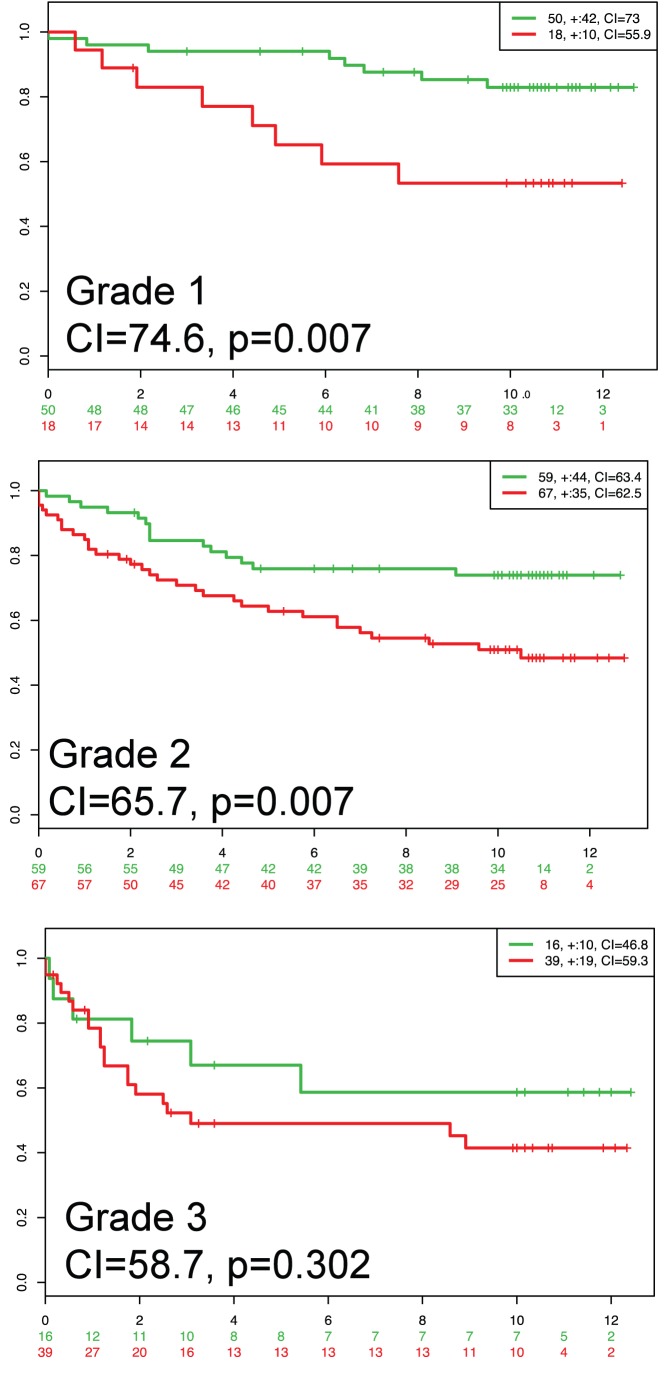
Kaplan-Meier curves and performance of the OncoTypeDX biomarker in the breast cancer Ivshina dataset across three tumor grades. Legends as in Figure 3.

As an example for testing one biomarker in several datasets, we used the 16 OncotypeDX genes [14]. OncotypeDX estimates a recurrence score that is mainly offered to early-stage, estrogen positive, lymph node negative breast cancers. The genes included are *AURKA*, *BAG1*, *BCL2*, *BIRC5*, *CCNB1*, *CD68*, *CTSL2*, *ERBB2*, *ESR1*, *GRB7*, *GSTM1*, *MKI67*, *MMP11*, *MYBL2*, *PGR*, and *SCUBE2* (*ACTB*, *GAPDH*, *GUSB*, *RPLP0*, and *TFRC* genes used as reference in the RT-PCR assay were not used here). To estimate the score, OncotypeDX uses a weighting algorithm equivalent to a weight multiplied by corresponding gene expression normalized by a reference [14]. In SurvExpress we used Cox fitting (as an approximation since gene expression data is not normalized to reference genes) in four breast cancer datasets (Table 3). Other settings were the maximum row average for genes with multiple probesets, and two risk groups split at the median of the prognostic index. To test the biomarker in several conditions, the datasets were chosen to reflect patients suitable for the test (Wang [27] and Ivshina [26]), patients with partial information besides different event (TCGA [25]), and patients without clinical information (Kao [15]). The results shown in Figure 3 and summarized in Table 4 suggest that, overall, Oncotype DX can separate significantly low- and high-risk groups in the four datasets tested. Moreover, satisfactory indexes of concordance and areas under the ROC curve were obtained. These results can be obtained using SurvExpress in a few minutes. To demonstrate the analytical features of SurvExpress, we also performed the survival evaluation stratifying the samples using the tumor grades provided by authors (AJCC Stage in the TCGA dataset and grade in the Ivshina dataset). Representative results for the Ivshina dataset are shown in Figure 4. The figure suggests that the performance, given by the concordance index and log-rank test for risk groups, decreases along grade. Results for the TCGA dataset are shown in the Tutorial available in the SurvExpress web site.

**Table 3:** Datasets and clinical for the OncotypeDX example.

Dataset	Platform	Samples/Censored	ER+/−	LN+/−	Outcome
**Breast Invasive Carcinoma TCGA ** [**25**]	Agilent	502/437	388/109		Survival
**Kao Huang Breast GSE20685 ** [**15**]	Affymetrix	327/244			Metastasis
**Ivshina Miller Breast GSE4922 ** [**26**]	Affymetrix	249/160	211/34	81/159	Recurrence
**Wang Foekens Breast GSE2034 ** [**27**]	Affymetrix	286/179	209/77	0/286	Recurrence

ER and LN stand for Estrogen Receptor and Lymph Node respectively.

**Table 4:** Results of the Oncotype DX in four breast cancer datasets.

Dataset	GenesFound	ResponseTime*	Risk GroupspValue	CI	DEG betweenRiskGroups	SurvivalROC**
**TCGA**	16	9.1 s	2.9e-7	72.2	11	0.74
**Kao GSE20685**	16	6.1 s	2.0e-5	69.1	16	0.69
**Ivshina GSE4922**	16	6.0 s	5.0e-6	68.7	13	0.70
**Wang GSE2034**	16	6.0 s	1.1e-7	69.1	13	0.73

CI stands for Concordance Index. DEG means differential expressed genes.

* Response time of the results page.

** SurvivalROC was estimated around time = 6 years, curves took one order of magnitude more than the response time shown.

#### Comparison of two lung cancer biomarkers

For non-small-cell lung cancer (NSCLC), at least 16 biomarkers have been proposed [16]. Here we compared two biomarkers proposed for survival of NSCLC that attempt to predict the same event (survival) and use a similar number of genes; however, the genes are different. The first NSCLC biomarker was proposed by Boutros *et al.* [17] and contains the following genes: *STX1A*, *HIF1A*, *CCT3*, *HLA-DPB1*, *RNF5*, and *MAFK*. The second NSCLC biomarker was proposed by Chen *et al.* [18] and contains the genes *DUSP6*, *MMD*, *STAT1*, *ERBB3*, and *LCK*. Therefore, it is of clinical interest to compare their performance. For this, we performed an analysis in SurvExpress using the maximum row average for genes with multiple probesets, two risk groups by prognostic index median, and Cox fitting. We used a special lung meta-base build in our research group, which is composed of more than 1,000 samples obtained from six authors (Bild [19], Raponi [20], Zhu [21], Hou [22], NCI [23], Okayama [24]), equivalent Affymetrix gene expression platform, and containing all biomarker genes.

**Figure 5:**
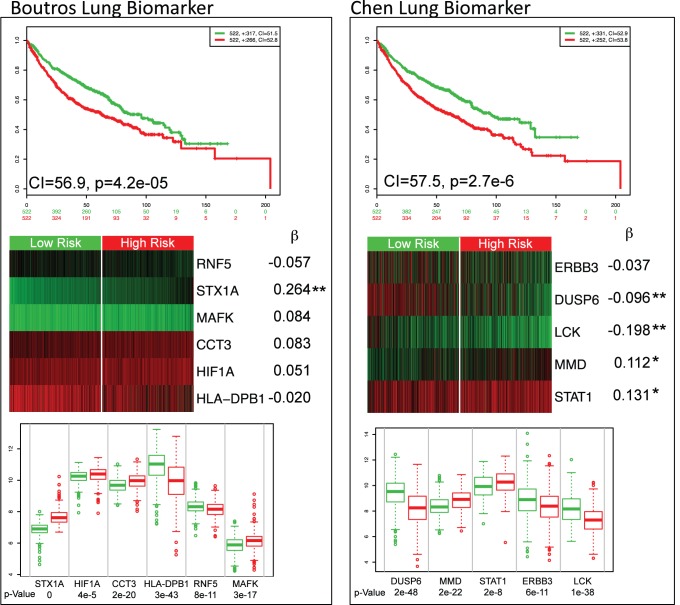
Performance and representation the two NSCLC biomarkers. Kaplan-Meier curves as in Figure 3. Heat map shows the expression of each gene (rows) along samples (columns) in risk groups. Low expression is represented in green grades and high expression in red grades. Corresponding beta coefficients from the Cox fitting is shown. Two stars (**) marks genes whose fitting p-value <0.05, one star (*) for marginal significant genes having p-value <0.10, and no stars for genes whose p-value is >0.1. Box plots compare the difference of gene expression between risk groups using a t-test.

**Figure 6:**
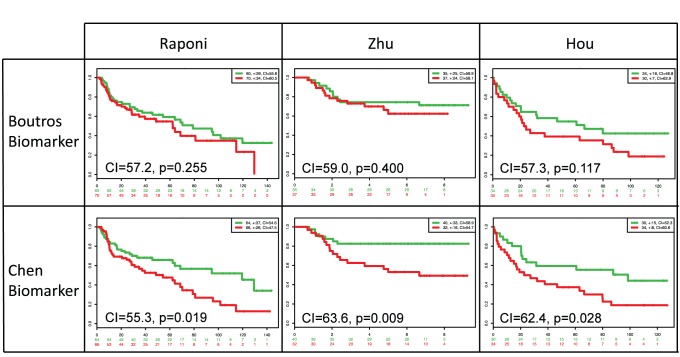
Comparison of Kaplan-Meier curves of the two NSCLC biomarkers in three representative lung cancer databases. Legends as in Figure 3.

The results show that both biomarkers are able to separate risk groups characterized by differences in their gene expression (see Kaplan-Meier and box plots respectively in Figure 5). Nonetheless, the p-value of the risk group separation, the concordance index, and the significance of the coefficients were slightly better in the Chen biomarker. To analyze the biomarkers more deeply, we tested the biomarker per database author using the SurvExpress stratification functionality (this can also be achieved performing a SurvExpress analysis per author dataset). The results for the six authors are summarized in Table 5. Three representative examples are shown in Figure 6. The results show that the Boutros biomarker fails in four datasets (the log-rank test of the difference in risk groups is not significant) while the Chen biomarker works better in almost all datasets. In summary, these results suggest that the performance of Chen biomarker is superior.

**Table 5:** Datasets and results of the Boutros and Chen biomarkers for the lung cancer example.

Dataset	Samples/Censored	Boutros p-RiskGroups	BoutrosOverall CI	Chen p-RiskGroups	ChenOverall CI
**Raponi Beer GSE4573 ** [**20**]	130/63	0.255	57.2	0.019	55.3
**Bild Nevins GSE3141 ** [**19**]	108/50	0.027	59.4	0.023	57.6
**Zhu Tsao GSE14814 ** [**21**]	72/49	0.401	59.0	0.009	63.6
**Hou Philipsen GSE19188 ** [**22**]	64/23	0.771	54.5	0.028	62.4
**Directoŕs Challenge Consortium NCI ** [**23**]	444/207	0.001	58.2	0.001	60.3
**Okayama Kohno GSE31210 ** [**24**]	226/191	0.222	59.1	0.006	66.1

p-Risk Groups column show the p-value of the equality between survival curves among risk groups.

## Conclusion

Compared with other tools, SurvExpress is the largest and the most versatile free tool to perform validation of multi-gene biomarkers for gene expression in human cancers. The analysis requires only the list of genes and can be performed in approximately one minute per dataset. Common applications for testing the performance of biomarkers include the evaluation of a biomarker in other populations or clinical status and the comparison of competing biomarkers. We have shown these two applications of SurvExpress comparing the performance of a breast cancer biomarker in several datasets, including tumor grades, and determining the best biomarker out of two alternative lung cancer biomarkers. We conclude that SurvExpress is a valuable and comprehensive web tool and cancer database with clinical outcomes tailored to rapidly evaluate gene expression biomarkers.
